# Supplementary material for: Self-rolling of vanadium dioxide nanomembranes for enhanced multi-level solar modulation
Source: Nat Commun. 2022 Dec 19;13:7819. doi: 10.1038/s41467-022-35513-w (PMC9763237; doi:10.1038/s41467-022-35513-w)
Supplement: Supplementary file 1 — Supplementary Information [file 41467_2022_35513_MOESM1_ESM.pdf]

Supplementary Information for

Self-rolling of vanadium dioxide nanomembranes for enhanced multi-level solar modulation

Xing Li[1,2,3+], Cuicui Cao[4,5+], Chang Liu[1,2,3], Wenhao He[6], Kaibo Wu[1,2], Yang Wang[1,2,3], Borui Xu[1,2], Ziao Tian[7], Enming Song[3,8], Jizhai Cui[1,2,3], Gaoshan Huang[1,2,3], Changlin Zheng[6], Zengfeng Di[7], Xun Cao[4,5*], Yongfeng Mei[1,2,3,8*]

1 Department of Materials Science & State Key Laboratory of ASIC and Systems, Fudan University, Shanghai 200438, People's Republic of China

2 Yiwu Research Institute of Fudan University, Yiwu 322000, Zhejiang, People's Republic of China

3 International Institute of Intelligent Nanorobots and Nanosystems, Fudan University, Shanghai 200438, People's Republic of China

4 State Key Laboratory of High Performance Ceramics and Superfine Microstructure, Shanghai Institute of Ceramics, Chinese Academy of Sciences, Shanghai 200050, People's Republic of China

5 Center of Materials Science and Optoelectronics Engineering, University of Chinese Academy of Sciences, Beijing 100049, People's Republic of China.

6 State Key Laboratory of Surface Physics and Department of Physics, Fudan University, Shanghai 200433, People's Republic of China

7 State Key Laboratory of Functional Materials for Informatics, Shanghai Institute of Microsystem and Information Technology, Chinese Academy of Sciences, Shanghai 200050, People's Republic of China

8 Shanghai Frontiers Science Research Base of Intelligent Optoelectronics and Perception, Institute of Optoelectronics, Fudan University, Shanghai 200438, People's Republic of China

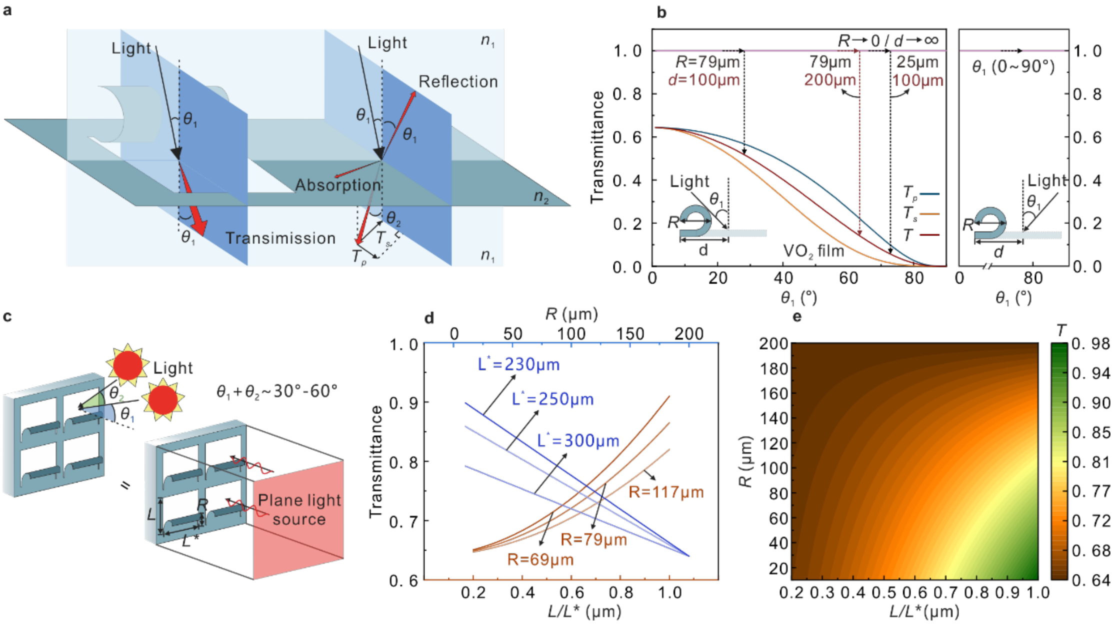

Supplementary Figure 1: **Particularity of rolled-up SW to the incident angle of the sun.** **a** Schematic of rolled-up SW and planar VO_2 NM for light transmission. **b** Transmittance of VO_2 SW with incident angle calculation by Fresnel formula. The left part presents the incident direction of sunlight in the same as the rolled-up direction and the right region in the opposite direction. **c** Schematic of sunlight incident on the rolled-up SW is equal to plane light incident regardless of the sun's incident angle. **d** Transmittance of the rolled-up SW with a single variable (radius, R or periodicity, L/L^*). **e** Transmittance of rolled-up SW as a function of R ($1/K$) and L/L^*. It indicates that the solar modulation of the rolled-up SW is only related to the structure size, and the change of the solar incidence angle has no influence.

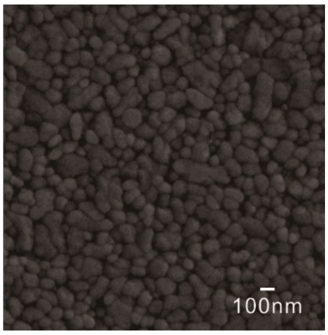

Supplementary Figure 2: **SEM image of VO_2 NM.** The SEM image shows that the surface morphology of the VO_2 NM is flat and the crystal grain size is about 80 nm in uniform distribution, which facilitates uniform transmission of sunlight.

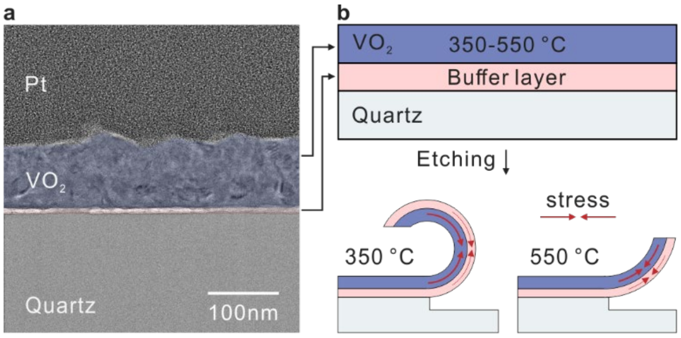

Supplementary Figure 3: **Stress gradients introduced into VO_2 NM. a** TEM image of the cross-section of VO_2 NM. **b** Schematic representations of rolling-up processes of single-component VO_2 nanomembranes. The red arrows qualitatively represent contraction of the layers after the NM released. The difference in these components leads to a lattice mismatch between the upper pure VO_2 NM and buffer layer, which creates a stress gradient in the vertical direction. When the NM with the vertical stress gradient is released from the substrate, the larger contraction of the upper part of the NM compared to the lower part creates a net bending force with the direction away from the substrate. At the same time, for NMs with same thickness, different growth temperatures enable the preparation of VO_2 NM with different initial stress gradients due to the difference in thermal expansion coefficients, which leads to formation of rolled-up VO_2 NM with different curvatures.

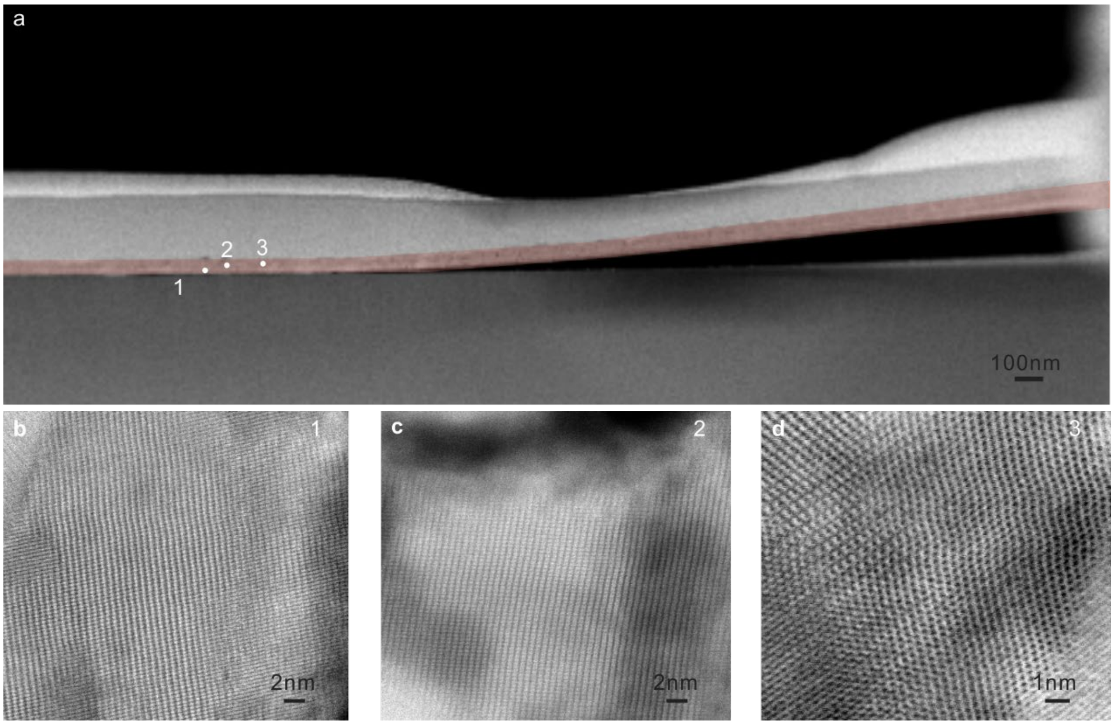

Supplementary Figure 4: **STEM images of VO_2 NM at different positions. a** Low-resolution STEM image of VO_2 NMs. **b-d** High-resolution STEM image at different positions corresponding to the (**a**). STEM images of different positions show good crystallinity.

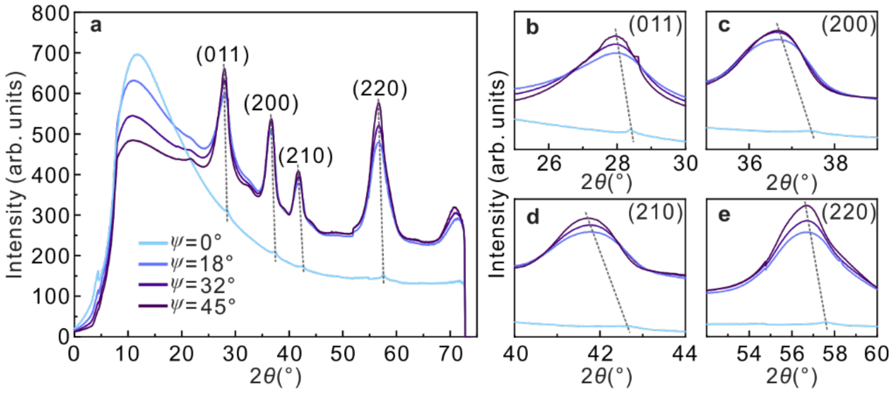

Supplementary Figure 5: **XRD patterns of VO_2 NM. a** XRD patterns measured by small-angle synchrotron radiation XRD with different incidence angles (ψ). **b-e** Corresponding enlarged spectra show individual VO_2 peaks. With the increase of ψ, the peak intensity of amorphous quartz around 12° decreases, while the characteristic peak intensity of VO_2 increases, and the peak position of VO_2 shifts to a smaller angle. It indicates that the initial internal strain gradient is introduced into the VO_2 NM which can be accurately calculated by a series of formulas (Supplementary Table 1, 2 and Note 2).

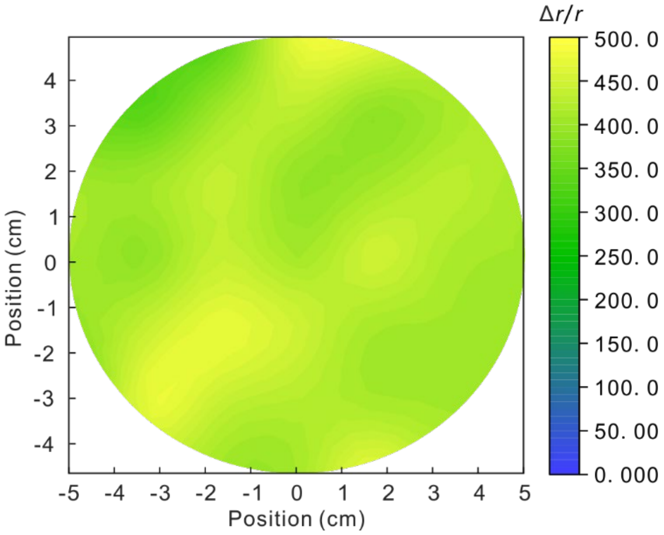

Supplementary Figure 6: **Map of 4-inch VO_2 resistivity change ratio.** More than 89% of the wafer area have the $\Delta r/r$ >350, demonstrating high yield, good uniformity and excellent quality of wafer-scale VO_2 NM.

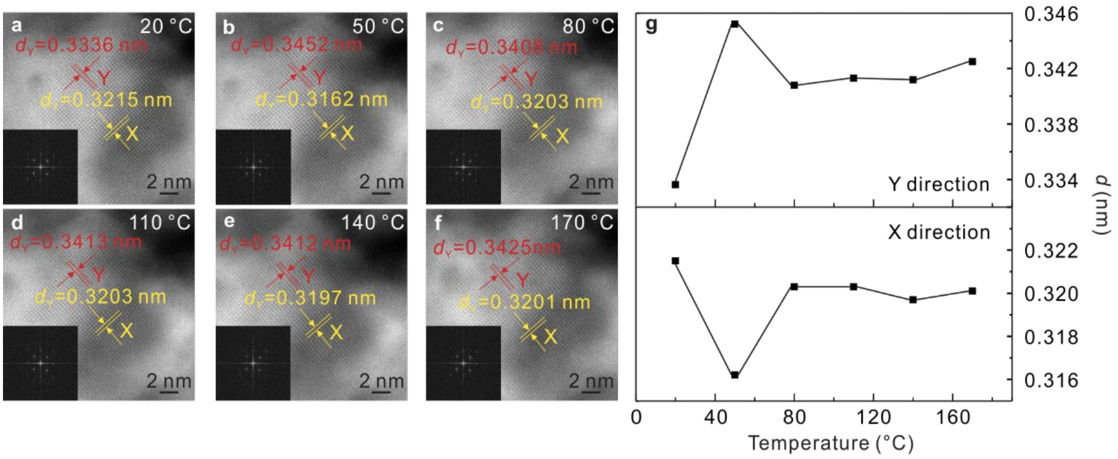

Supplementary Figure 7: **In-situ STEM image and corresponding FFT image of VO_2 NM during heating.** **a** 20 ºC, **b** 50 ºC, **c** 80 ºC, **d** 110 ºC, **e** 140 ºC, **f** 170 ºC. **g** Interplanar spacing (d) in two directions changes with temperature. The d of the VO_2 has a regular and obvious change during the heating process, which infer the occurrence of the phase transition and the resulting strain change. Y-direction is corresponding to the [0-12] zone axis, and X-direction represents the [012] zone axis. Here, the value of d shows an obvious sudden change during heating before reaching τ_c and tends to a relatively stable value after the phase transition. Since the internal stress existing in the NM will accumulate certain elastic potential energy, the phase transition will destroy the equilibrium state and release the potential energy. After the phase transition, the release of the potential energy will make the lattice structure forms a new equilibrium state and stabilize d.

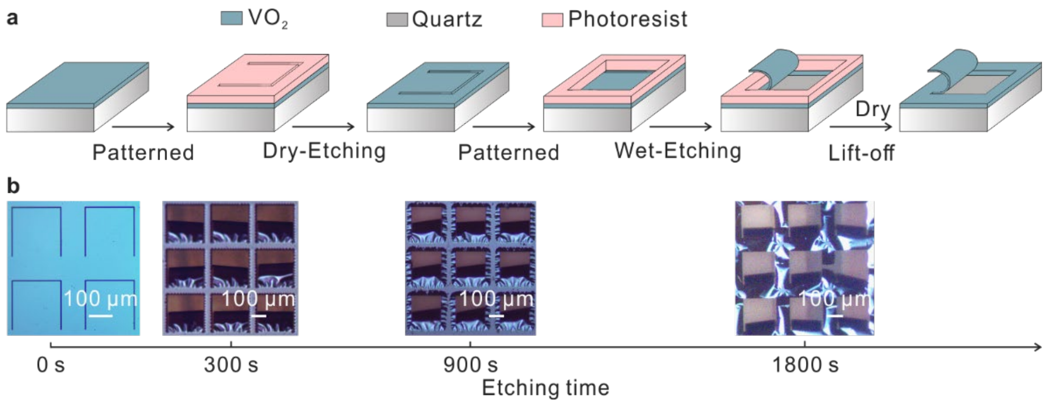

Supplementary Figure 8: **Fabrication process for SWs with very small intervals in a rolled-up structure. a** The schematic of fabrication process using rolled-up nanotechnology. **b** Microscope images of sample with different etching times. In HF etching step, the quartz in the framework regions will also be gradually etched and the VO_2 NMs in the framework regions are also partially release from the substrate. It is worth noting that the framework NMs are interconnected and therefore rolling is unlikely realized in these framework regions with short etching time. In order to further fix NMs in these regions, in the fabrication process of our experiment, we perform an additional step of lithography before wet-etching to cover the framework with a thick photoresist layer, which helps to maintain the framework geometry. The experimental results also confirm the effectiveness of the method, especially for short etching time. It can be seen that the framework is intact until the etching time reaches 1800 s, which is much longer than normal etching time of ~300 s.

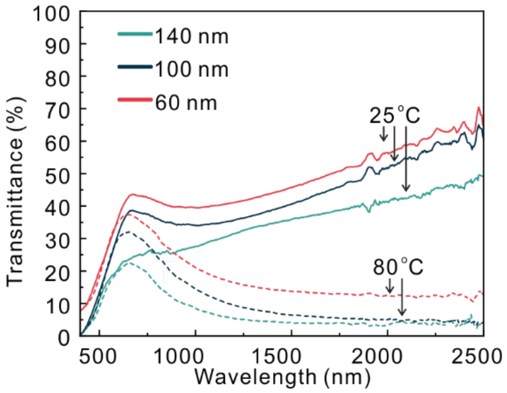

Supplementary Figure 9: **Transmittance spectra of the VO_2 NMs with different thicknesses.** The 100 nm VO_2 NM has the best solar modulation during phase transition.

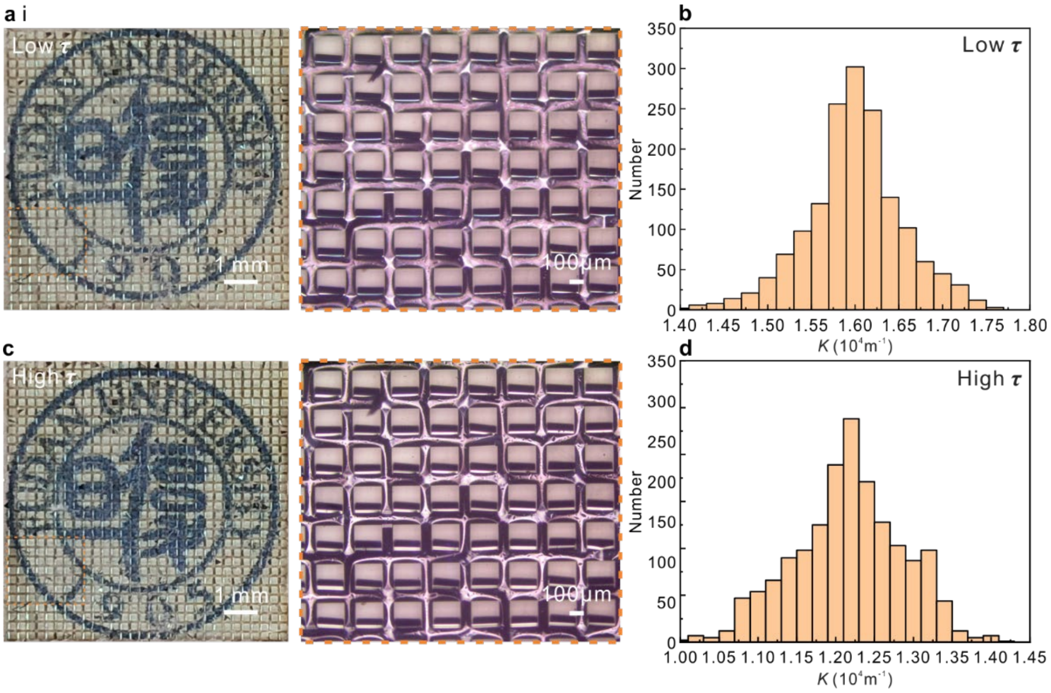

Supplementary Figure 10: ***K* statistics for sample i.** **a** Photograph (left) and corresponding optical microscope image (right) of sample i at low τ. **b** Statistics of sample i at low τ. **c** Photograph (left) and corresponding optical microscope image (right) of sample i at high τ. **d** Statistics of sample i at high τ.

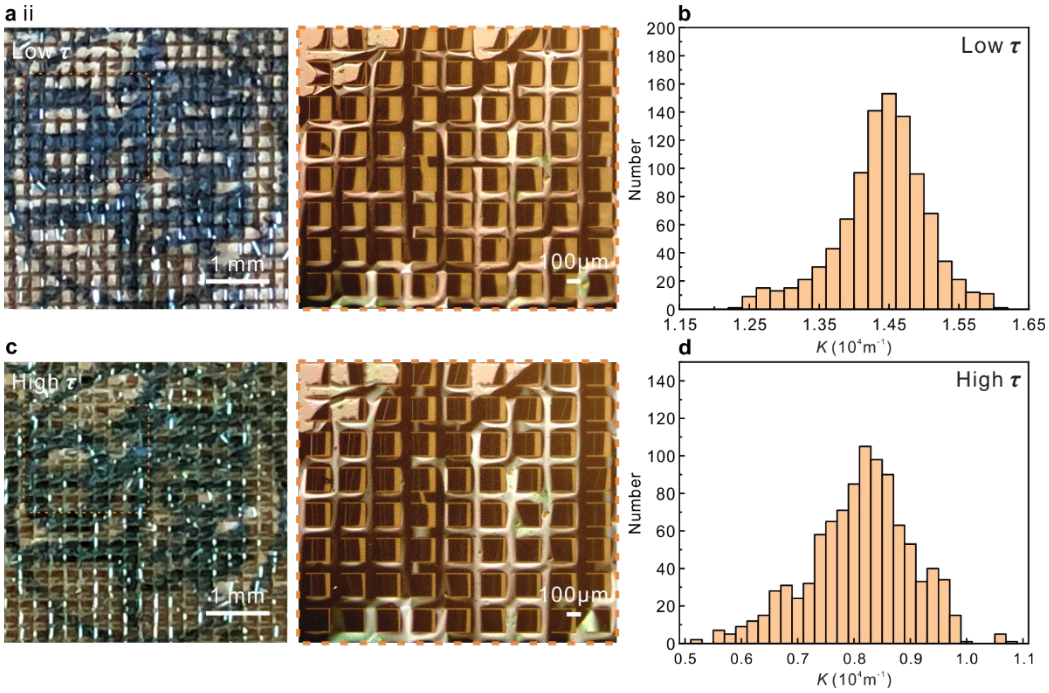

Supplementary Figure 11: ***K* statistics for sample ii. a** Photograph (left) and corresponding optical microscope image (right) of sample ii at low τ. **b** Statistics of sample ii at low τ. **c** Photograph (left) and corresponding optical microscope image (right) of sample ii at high τ. **d** Statistics of sample ii at high τ.

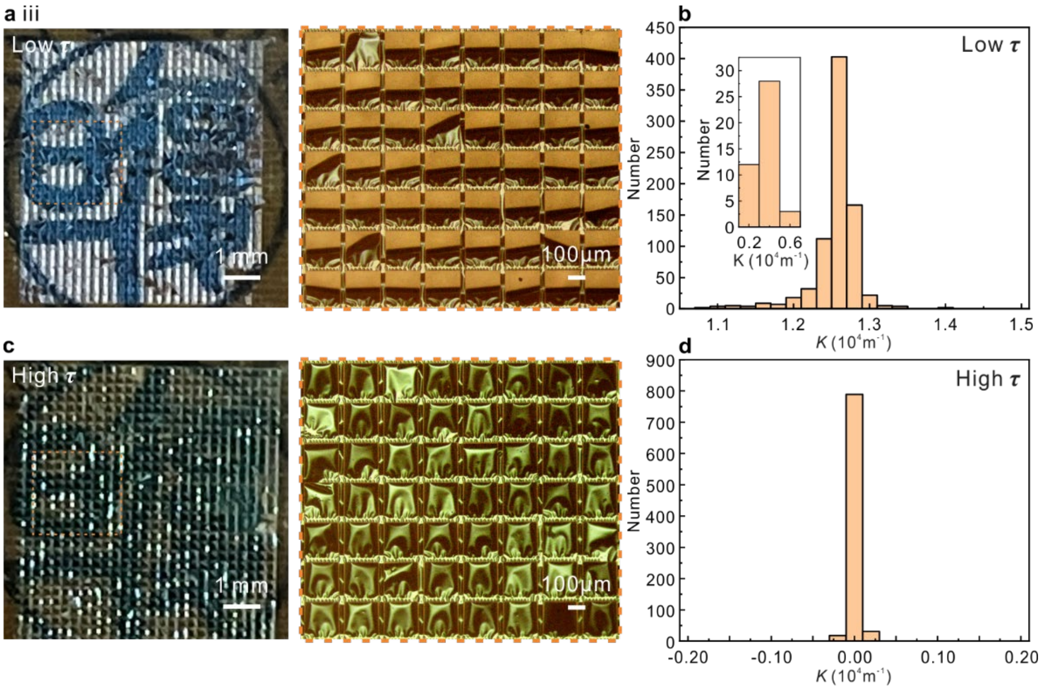

Supplementary Figure 12: ***K* statistics for sample iii. a** Photograph (left) and corresponding optical microscope image (right) of sample iii at low τ. **b** Statistics of sample iii at low τ. **c** Photograph (left) and corresponding optical microscope image (right) of sample iii at high τ. **d** Statistics of sample iii at high τ.

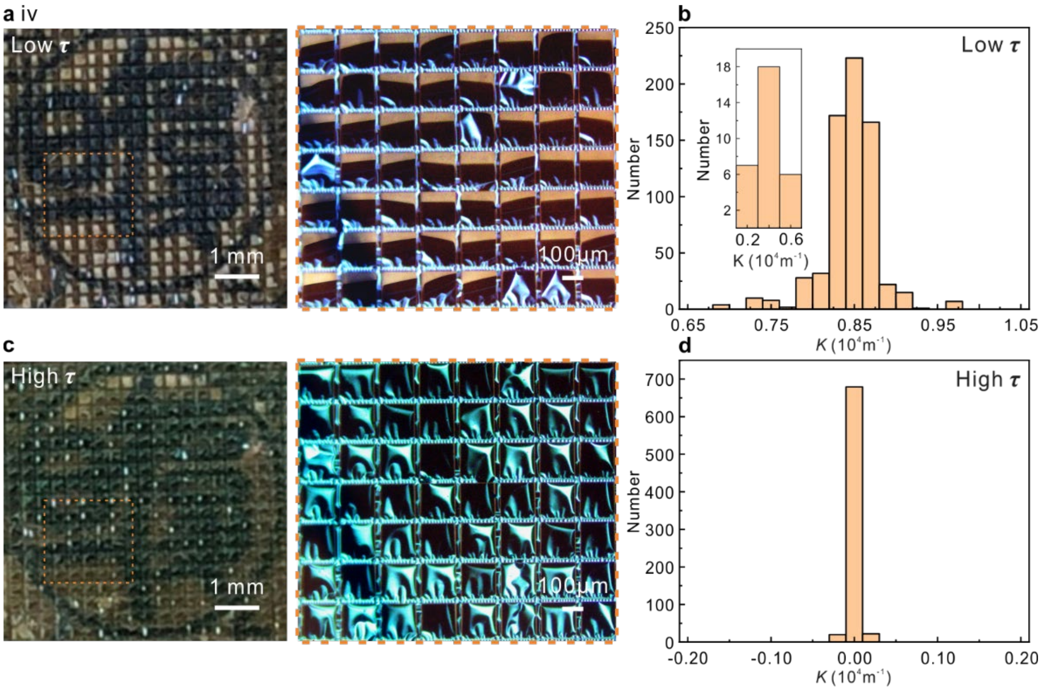

Supplementary Figure 13: ***K* statistics for sample iv. a** Photograph (left) and corresponding optical microscope image (right) of sample iv at low τ. **b** Statistics of sample iv at low τ. **c** Photograph (left) and corresponding optical microscope image (right) of sample iv at high τ. **d** Statistics of sample iv at high τ.

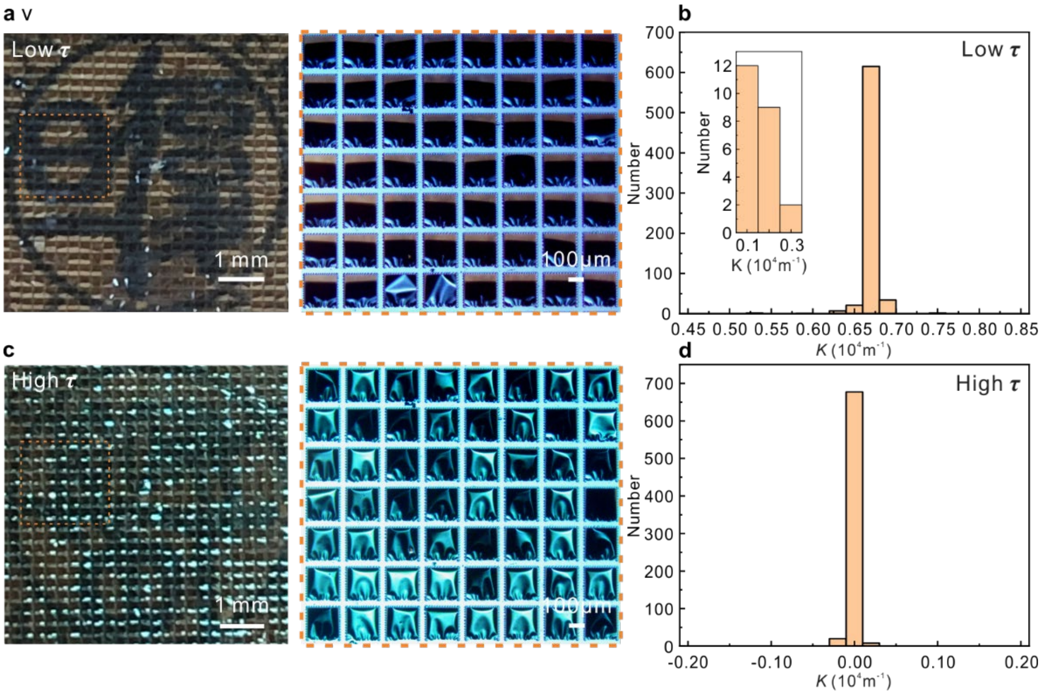

Supplementary Figure 14: ***K* statistics for sample v. a** Photograph (left) and corresponding optical microscope image (right) of sample v at low τ. **b** Statistics of sample v at low τ. **c** Photograph (left) and corresponding optical microscope image (right) of sample v at high τ. **d** Statistics of sample v at high τ.

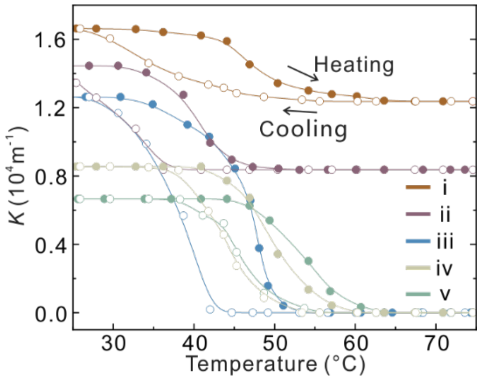

Supplementary Figure 15: **Curvatures of different rolled-up VO_2 samples as a function of temperature.** The rolled-up microstructures have a large curvature (K) change during the phase transition, and the statistics of the K show that the internal stress released by the rolling leads to the decrease of the phase transition temperature (τ_c).

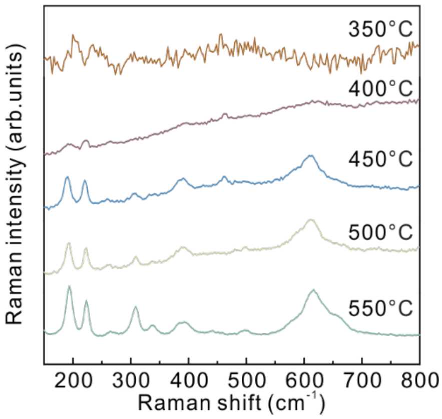

Supplementary Figure 16: **Raman spectra of VO_2 NM samples deposited at different temperatures excited by laser with wavelength of 532 nm.** The VO_2 NMs with low growth temperature have relatively weak Raman peak. In contrast, the relative peak intensities improve significantly when the growth temperature is increased to 450 °C. This proves the poor crystalline quality of the VO_2 NM deposited at low temperature.

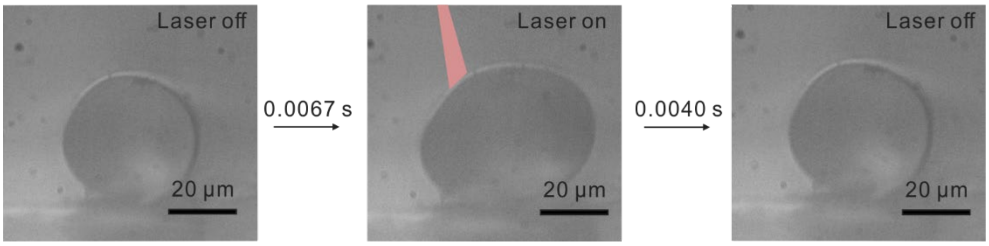

Supplementary Figure 17: **Side views of 808 nm laser-excited rolled-up structure with phase transition.** We used a high-speed camera to record the actuation process of a laser-heated rolled-up structure, which took 6.7 ms from the laser “on” to the completion of the rolling process, and 4 ms to for the structure to recover its original shape after the laser was turned off.

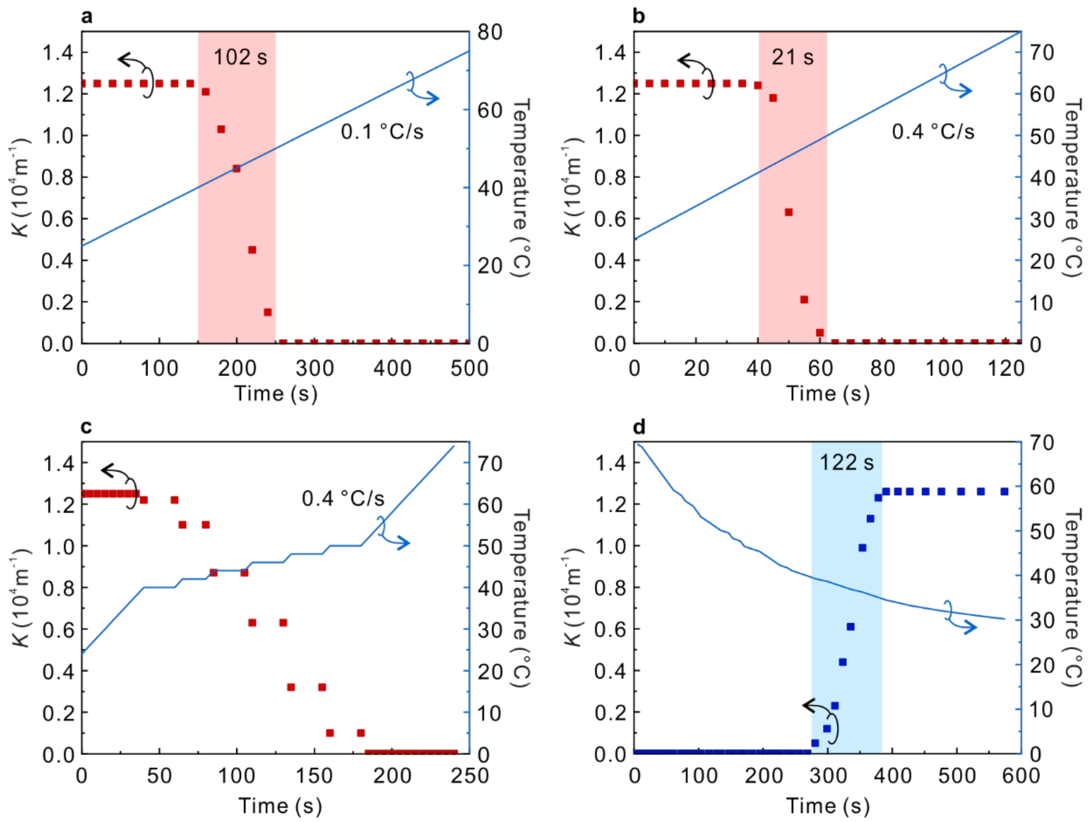

Supplementary Figure 18: ***K* of sample iii in heating or cooling process. a** *K* of sample iii as a function of time in heating process (0.1 °C/s). **b** *K* of sample iii as a function of time in heating process (0.4 °C/s). **c** *K* of sample iii as a function of time in stepwise heating process. **d** *K* of sample iii as a function of time in cooling process. **a** shows the *K* versus time curve for a heating rate of 0.1 °C/s, the deformation time is about 102 s. When the heating rate is increased to 0.4 °C/s, the deformation time decreases to 21 s (**b**). **c** shows the stepwise heating process, start at 40 °C and wait 10 s for every 2 °C rise. It is found that when the temperature stabilizes at any temperature between 40 °C and 50 °C, the rolled-up SW is corresponded to a stable *K*. During the natural cooling process, deformation of rolled-up structure started at 40 °C and the structure restore the original *K* at 34 °C, and the recovery process taking approximately 122 s (**d**). These results show that although the deformation of the rolled-up structure is abrupt after the ambient temperature reaches the triggering temperature, the actual temperature change is slower leading to a gradual deformation.

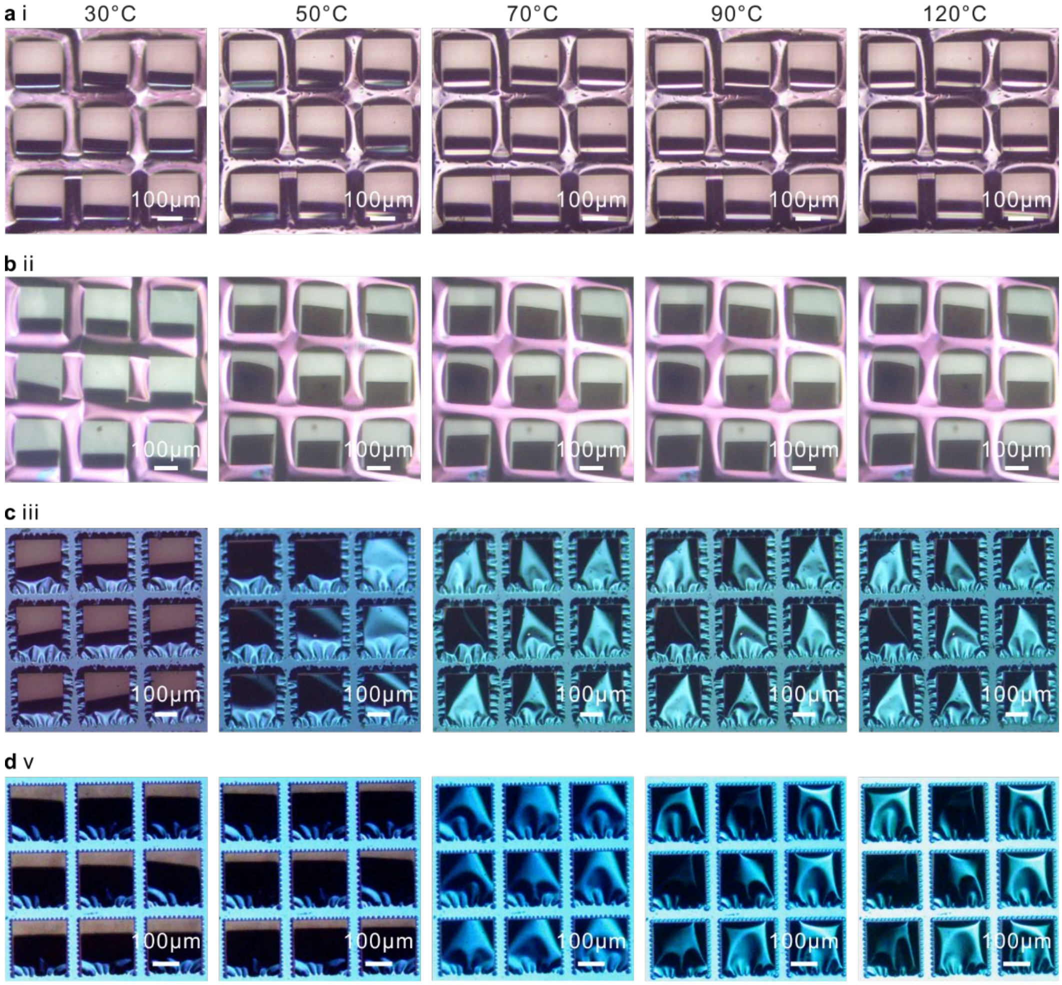

Supplementary Figure 19: **Structural changes in rolled-up samples during heating.** **a** Microscope image of sample i during heating. **b** Microscope image of sample ii during heating. **c** Microscope image of sample iii during heating. **d** Microscope image of sample v during heating. Each sample was subjected to continuous heating to 120 °C after the phase transition and it was found that continuous heating did not lead to observable structural changes.

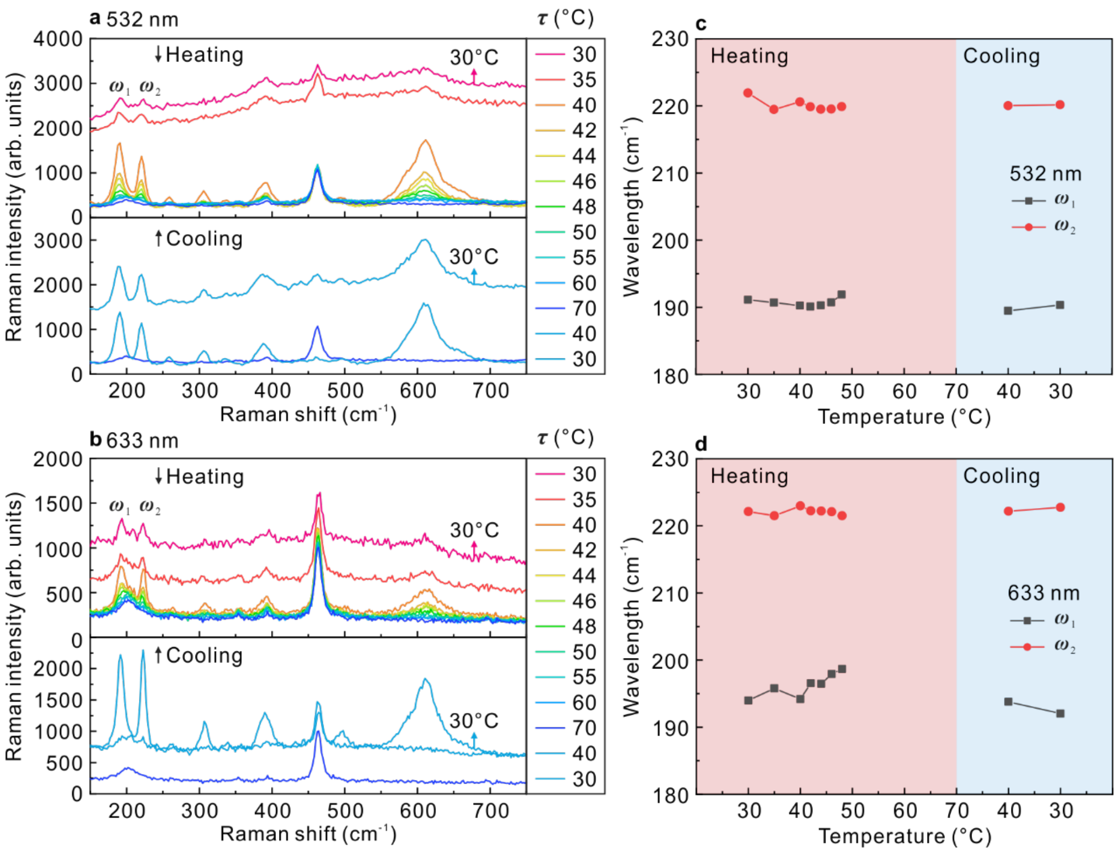

Supplementary Figure 20: **Raman spectra of a rolled-up structure measured at different temperatures. a** Raman spectra of a rolled-up structure measured in heating or cooling process excited by laser with wavelength of 532 nm**. b** Raman spectra of a rolled-up structure measured in heating or cooling process excited by laser with wavelength of 633 nm. **c** Raman peak positions (ω_1 and ω_2) as a function of temperature (laser: 532 nm). **d** Raman peak positions (ω_1 and ω_2) as a function of temperature (laser: 633 nm). Raman peak of VO_2 disappears at 50 °C and appears again while the temperature reduces to 40 °C. It can be visualised that the position of the peaks fluctuates within the tolerance range before the phase transition, indicating a trivial strain change in this stage. However, it is worth noting that characterization of the strain change during the phase transition by Raman is difficult because the metal phase of VO_2 demonstrates no Raman activity.

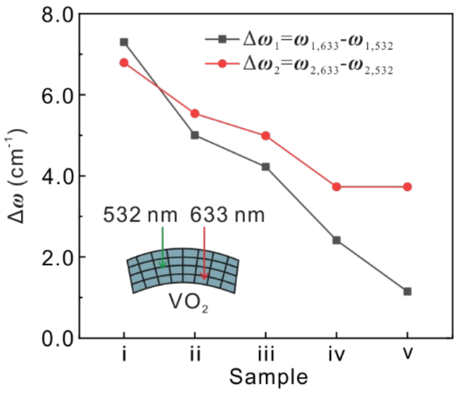

Supplementary Figure 21: **Raman peak shifts (Δω) of ω_1 and ω_2 in rolled-up SW samples obtained with different lasers wavelengths correspond to: i (350 °C), ii (400 °C), iii (450 °C), iv (500 °C) and v (550 °C).** The shifts of Raman peaks (Δω) excited by different lasers are summarized and both Δω_1 and Δω_2 reduce with increase growth temperature. According to these shifts, the initial strain gradient (Δε) along the radial direction are calculated (Supplementary Note S3).

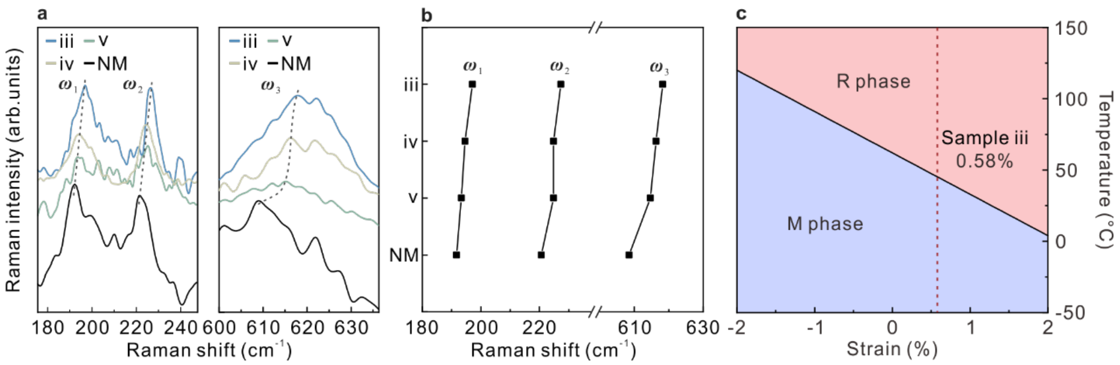

Supplementary Figure 22: **Raman peak shifts of different samples and Phase diagrams of VO_2. a** Raman spectra of sample iii, iv, v and VO_2 NM excited by lasers with wavelengths of 633 nm at room temperature. **b** Peak positions (ω_1, ω_2 and ω_3) determined from Lorentzian fitting of the spectra shown in (**a**). **c** Phase diagrams of VO_2. To further demonstrate the strain generation by rolling, we compared the Raman spectra of ω_1, ω_2 and ω_3 peaks of samples iii, iv, v and VO_2 NM under 633 nm laser (**a**) and their peak shifts are summarized in **b**. It can be seen that the changes of strain lead to a monotonic shift, and this result agrees with previous literature and the strain calculations are close to the actual test results in these articles. In phase diagrams of VO_2 (**c**), the red line is the strain of the sample iii. It shows that the calculated strain value and the actual τ_c are consistent with the phase diagram data.

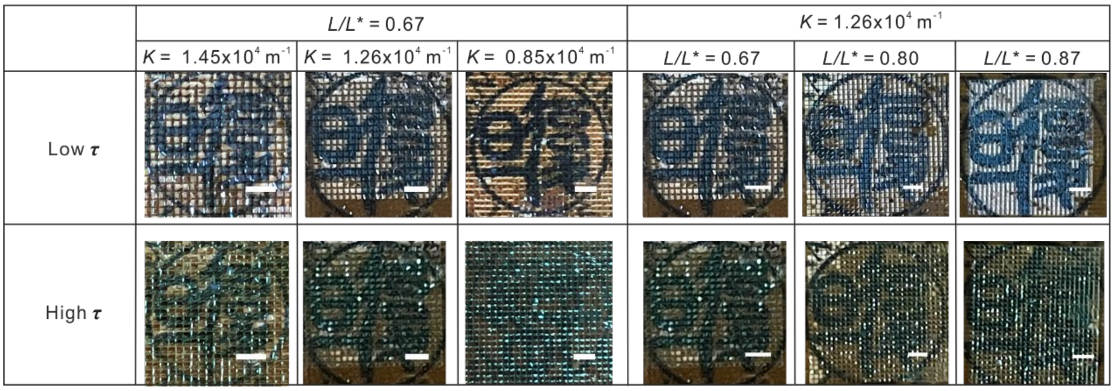

Supplementary Figure 23: **Photograph of rolled-up SWs.** Scale bar: 1 mm. Structural optimization has an obvious effect on enhancing the light transmittance. Under the condition that the maximum deformation of rolled-up SW is satisfied, the reduction of L/L^* can effectively enhance the light transmittance at low τ.

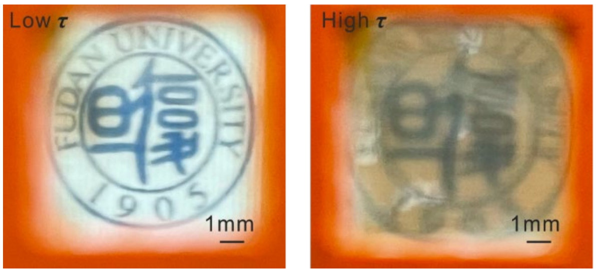

Supplementary Figure 24: **Photograph of the "Fudan" badge is observed through the rolled-up SW at different temperatures.** The badge is far away from the rolled-up SW. To demonstrate the excellent performance of rolled-up SW with a K of 1.26×10^4 m^{-1} and a L/L^* of 0.87 in light transmittance, the sample is placed on a heating plate with a small hole and the "Fudan" badge is placed 15 cm away from the sample. In corresponding photos, the sharpness of the badge drops significantly at high τ, while the color and shape of the badge are well displayed at low τ.

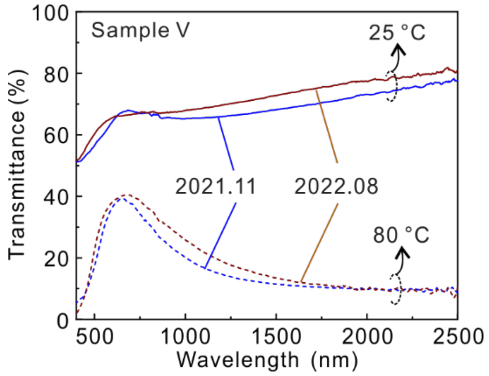

Supplementary Figure 25: **Transmittance spectra of the sample V after fatigue tests.** The rolled-up SW has good structural stability and deformation recyclability, and it can be used efficiently for long periods of time.

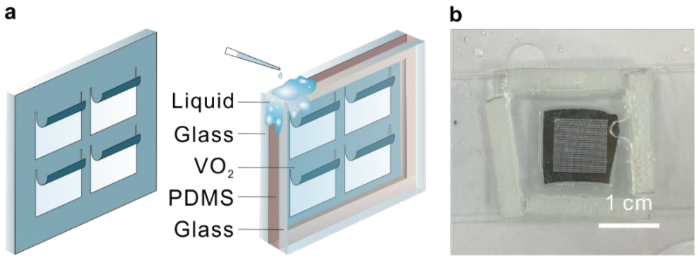

Supplementary Figure 26: **Encapsulating of rolled-up SW. a** Schematic of an encapsulated device. **b** Encapsulated rolled-up SW. We place the rolled-up SW sample in the center of normal glass, and the sample is surrounded by polydimethylsiloxane (PDMS) as a bracket to support for the upper glass. The PDMS is then cured at high temperature to form a completely sealed cavity, and thus the sample is insulated from water, wind, air and other factors that can cause damage to the rolled-up structure.

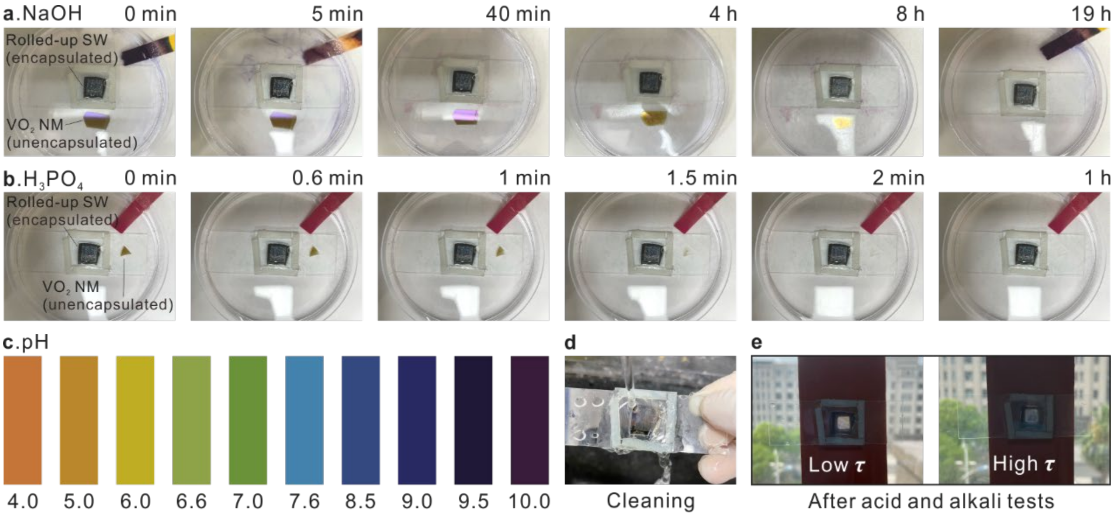

Supplementary Figure 27: **Encapsulated rolled-up SW test in extreme environments.** Encapsulated rolled-up SW and VO_2 NM placed in (**a**) NaOH and (**b**) H_3PO_4 solution. **c** Reference pH values. **d** The process of cleaning with flush of water flow between each test. **e** Light transmission modulation ability after acid and alkaline tests. These results indicate that the encapsulated rolled-up SW should be able to meet the requirements of the complex situations in practical environment.

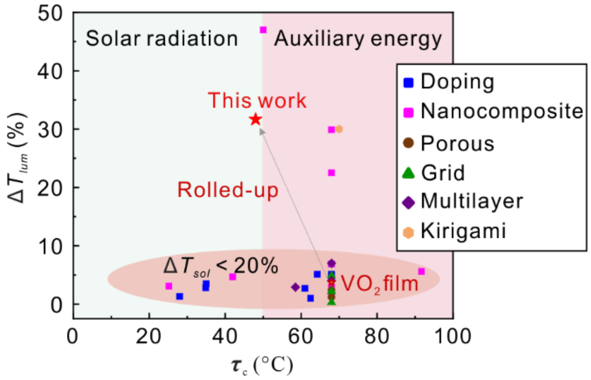

Supplementary Figure 28: **Comparison of this work with the best-reported experimental works regarding the ΔT_{lum} and τ_c[1-27].** ΔT_{lum} shows the change of visible light transmission during phase transition, which means visibility change of the outdoor scene. Generally, in order to ensure high transmission, this difference of transmittance should be minimized as much as possible, so that the window's basic skills can still be maintained based on energy-saving. Here, our rolled-up SW has a great transmittance at low τ and meets the minimum requirements of civil windows for lighting at high τ. The τ_c can be reduced to below 50 °C through strain engineering without doping, which provides the possibility for practical applications.

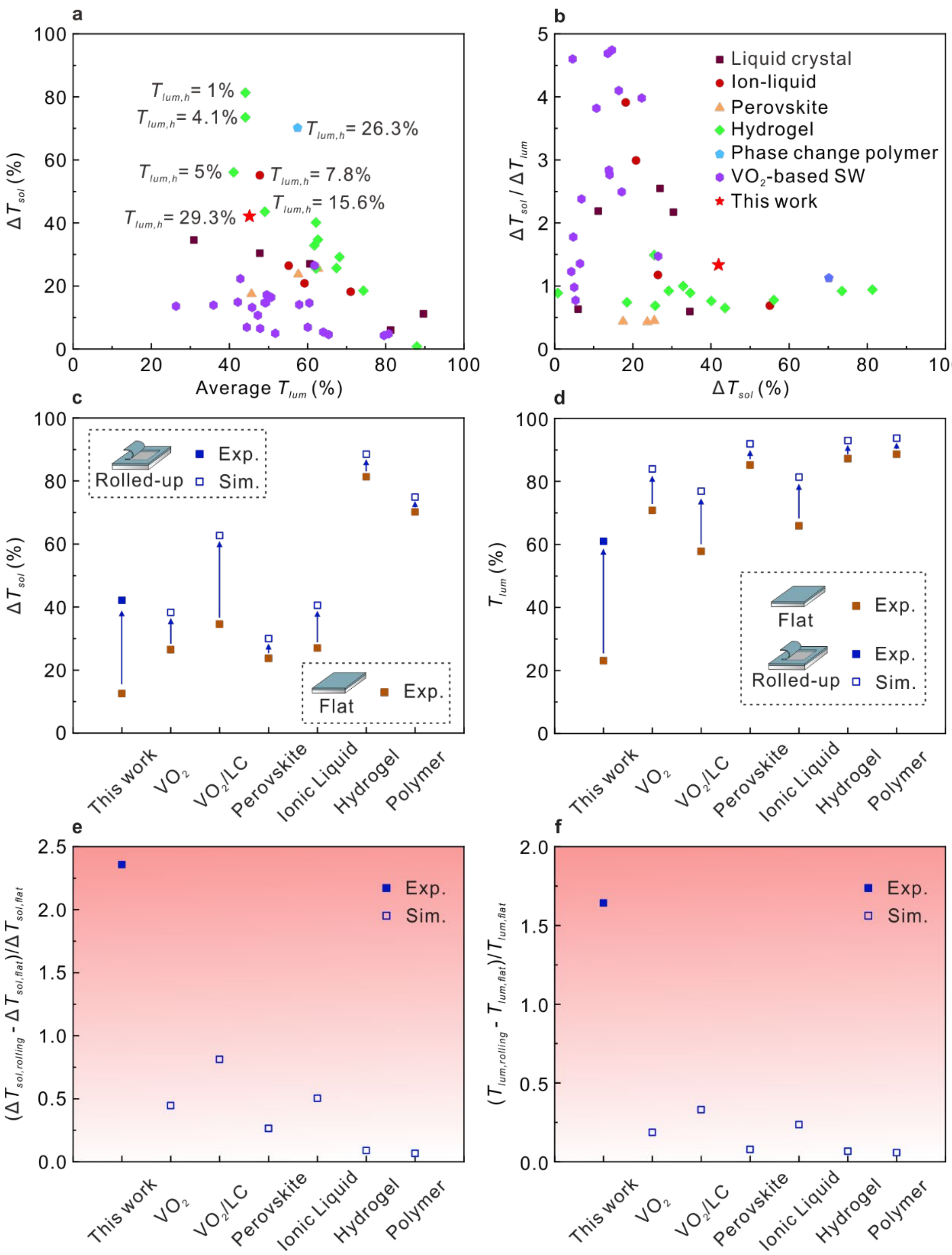

Supplementary Figure 29: **Comparison of this work with different materials-based SW works regarding the T_{lum} and ΔT_{sol}.** **a** Comparison of this work with the best-reported experimental works regarding the average T_{lum} and ΔT_{sol}. **b** Comparison of this work with the best-reported experimental works regarding the ΔT_{sol} and $\Delta T_{sol}/\Delta T_{lum}$. **c** Comparison of this work with simulated ΔT_{sol} enhancement due to rolling. **d** Comparison of this work with simulated T_{lum} due to rolling. **e** Comparison of ΔT_{sol} improved rates of this work and simulated other rolled-up structures made from different materials. **f** Comparison of T_{lum} improved rates of this work and simulated other other rolled-up structures made from different materials. Here, a more

comprehensive comparison is demonstrated (**a**). Hydrogels, ionic liquids, and phase change polymers-based SW have achieved very high ΔT_{sol}, but their T_{lum} at high τ is approaching 0 which means they are loss of lighting capacity. Therefore, obtaining high ΔT_{sol} means minimising light transmission at high temperatures. We further compare $\Delta T_{sol}/\Delta T_{lum}$ versus ΔT_{sol} for different materials (a larger $\Delta T_{sol}/\Delta T_{lum}$ means increasing ΔT_{sol} and smaller change in visible light transmission during two status). It can be seen that the normal SW which modulates the ΔT_{sol} by changing visible transmission will have relatively low $\Delta T_{sol}/\Delta T_{lum}$ (**b**). This comparison highlights the advantages of VO_2 SWs that improves the ΔT_{sol} without changing the lighting. Moreover, our rolled-up technology can be combined with different material systems to achieve further improvements on the performance of SW. In **c** and **d**, the rolled-up technique is supposed to be applied in different material systems (the calculation method is shown in Supplementary Note 5). Performance improvement rates of rolled-up SWs made from different materials are shown in **e** and **f**. The improvement rates for both ΔT_{sol} and T_{lum} in this work are much higher than those for other materials. The results indicate the advantages of the rolled-up technique in improving the performance of planar SWs, especially for films that are inherently with poor visible light modulation, or for planar structures that need to improve visible light transmission at low τ.

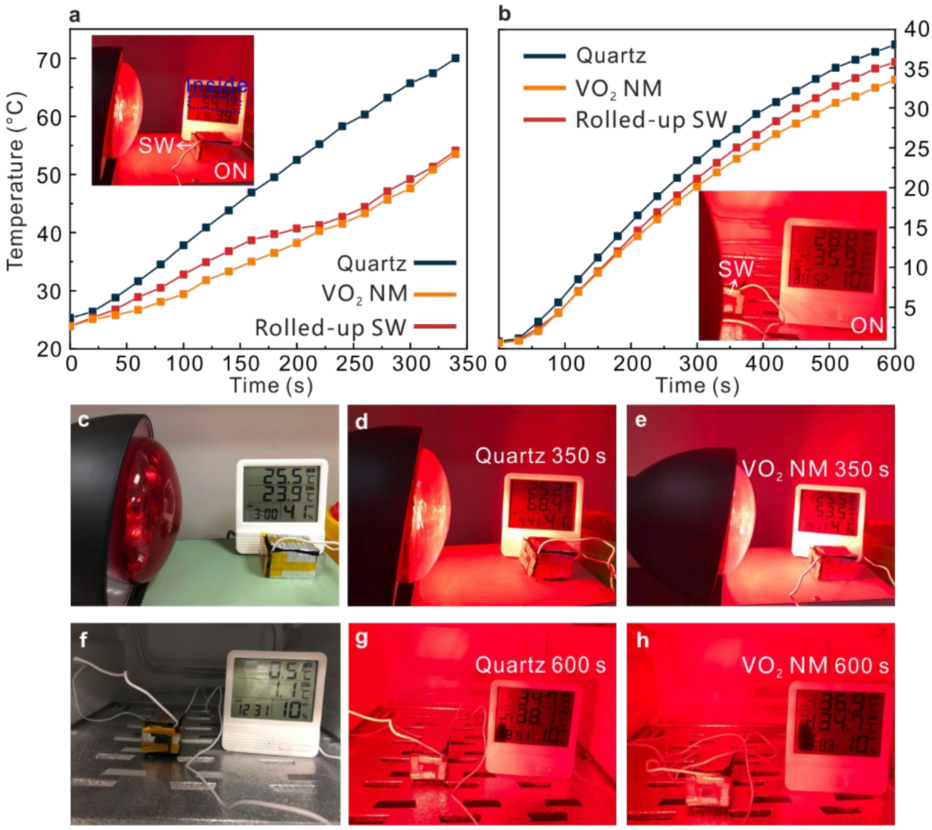

Supplementary Figure 30: **Comparison of the in-house temperature changes of different windows. a,** In-house temperature dependence on the irradiation time for quartz, VO_2 NM and the rolled-up SW. The inset shows the temperature sensor connects with the house model and IR light for heating (room temperature is about 25.5 °C). **b,** In-house temperature dependence on the irradiation time for quartz, VO_2 NM and the rolled-up SW. The inset shows the temperature sensor connects with the house model and IR light for heating (room temperature is about 0.5 °C). **c-e,** Photographs of quartz or VO_2 NM-installed house heated by IR light in on or off state (room temperature is about 25.5 °C). **f-h,** Photographs of quartz or VO_2 NM-installed house heated by IR light in on or off state (room temperature is about 0.5 °C). The house has two layers, the outer layer is glass for support, and the interlayer is an insulating sponge. The heat exchange of the house only depends on the radiation from a hollow that is on the house wall. For the quartz-installed house, the temperature rises to 70 °C in 350 s. However, the rolled-up SW and the VO_2 NM SW reach 51 °C after 350 s due to the phase transition. Interestingly, rolled-up SW has better light transmittance before the phase transition leading to a faster heating rate. When the house is put in the refrigerator and heated by the same heating lamp, the different windows-installed houses' temperature change trends are similar, and the temperature difference is about 3-4 °C. Compared with the VO_2 NM SW, the rolled-up SW shows a better heat exchange at low τ.

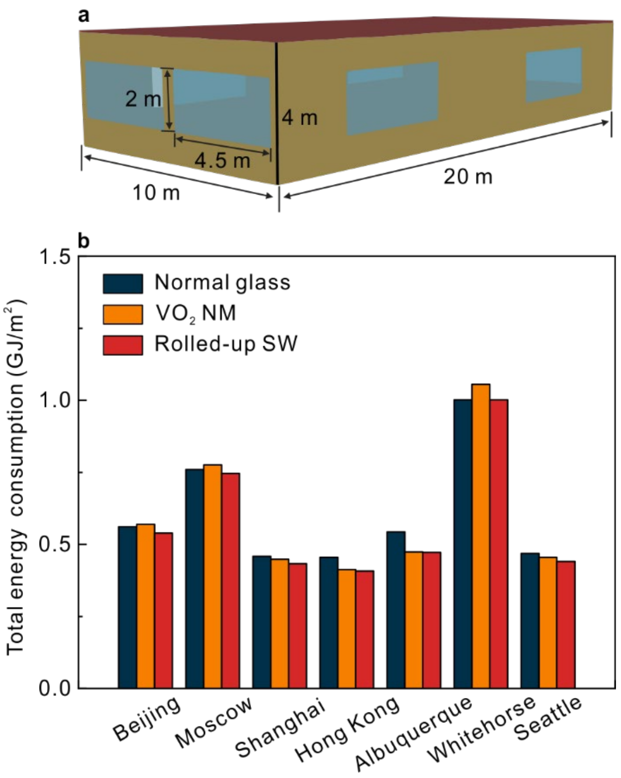

Supplementary Figure 31: **Simulation for energy-saving performance in buildings.** **a** The building model used in the simulation. **b** Detailed total energy consumption (E_c, include cooling, heating and lighting) in the building. A house model was built to simulate the energy-saving with installing different windows by EnergyPlus, the window-to-wall ratio is 0.3. Here, the τ_c was set at 24 ºC to better demonstrate the potential of the rolled-up SW in the future energy conditioning of the house. The calculated results clearly show that the rolled-up SWs provide energy savings in 7 cities whatever the climates are. The building model and simulation parameters are listed in Supplementary Table 5 and 6. A comparison of the simulated energy saving (E_{saving}) results for different smart windows is shown in Supplementary Table 7.

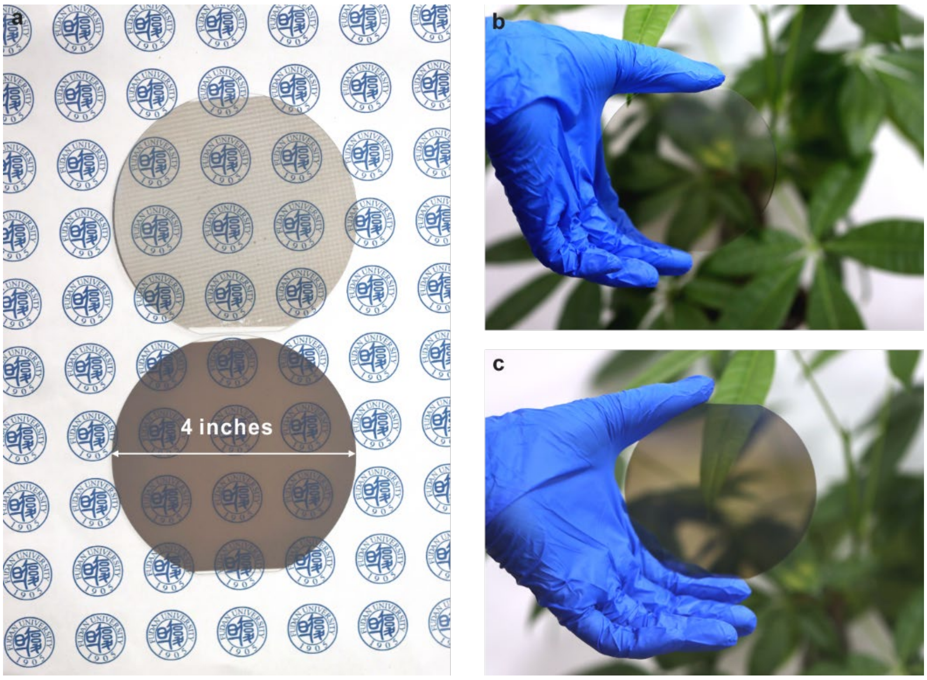

Supplementary Figure 32: **Wafer-scale VO_2 SW grown on a 4-inch quartz substrate.** **a** Comparison of patterned VO_2 NM wafer (4-inch) with unpatterned VO_2 NM wafer (4-inch) in transparency. **b** Photograph of patterned VO_2 NM wafer (4-inch). **c** Photograph of unpatterned VO_2 NM wafer (4-inch).

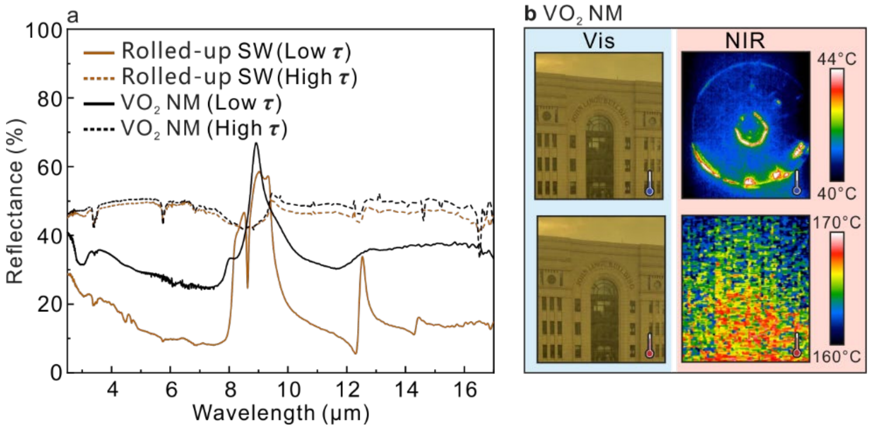

Supplementary Figure 33: **Reflectance spectra of rolled-up SW during the phase transition. a** Reflectance spectra of the VO_2 NM and rolled-up SW at different temperatures. It shows the reflectivity of the rolled-up SW ($K = 1.26\times10^4$ m^{-1}, $L/L^* =$ 0.87) and the VO_2 NM SW at different temperatures measured by Fourier transform infrared spectroscopy (FTIR). It can be seen that the reflectivity of rolled-up SW reduces a lot at low τ, and approaches that of the VO_2 NM at high τ. **b,** Photograph was taken by the camera through a window installed with VO_2 NM SW. Blue thermometers represent low τ and red thermometers represent high τ. The VO_2 NM cover has similarly visible and IR imaging to the rolled-up SW cover at high τ, but it shows a significant difference at low τ.

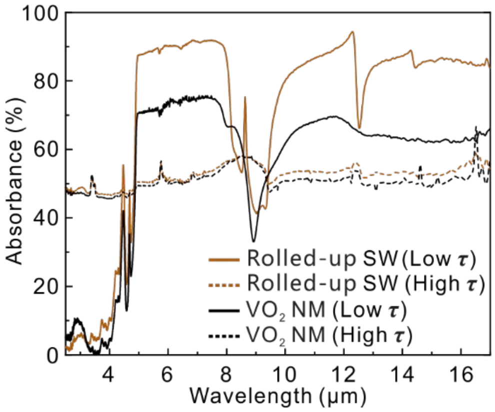

Supplementary Figure 34: **Absorption spectra of rolled-up SW during the phase transition.** Absorption spectra of VO_2 NM and rolled-up SW at different temperatures. It shows the absorption of the rolled-up SW ($K = 1.26\times10^4$ m^{-1}, $L/L^* = 0.87$) and the VO_2 NM at different temperatures by FTIR. It can be clearly seen that the absorption of rolled-up SW reduces a lot at low τ, and approaches that of the VO_2 NM at high τ. The wide range of emissivity variation in rolled-up SW can bring great practicality to the field of camouflage coating.

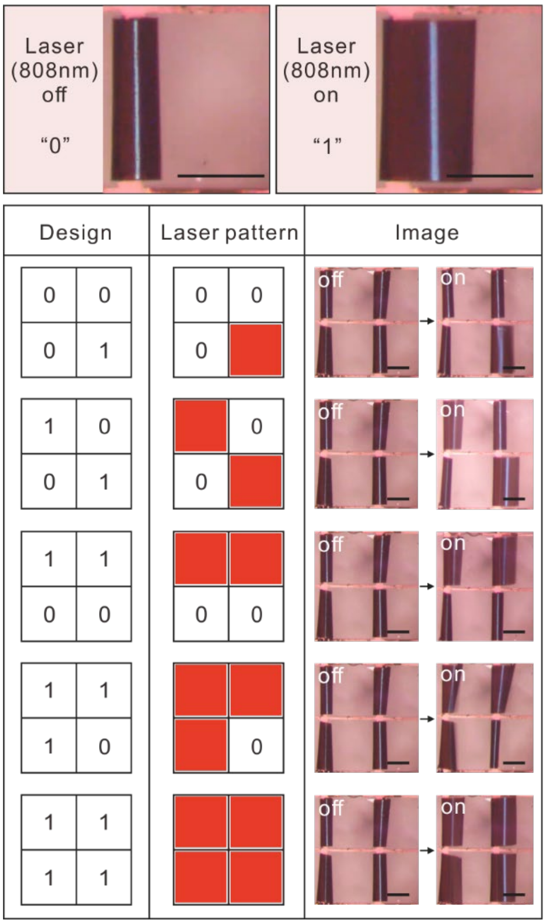

Supplementary Figure 35: **Fixed area actuation of rolled-up SW is controlled by the patterned laser for infrared imaging.** With a single rolled-up structure as a pixel, a specific laser pattern can realize the intelligent driving of single or multiple pixels. It shows that the rolled-up SW can not only work together in a large array but also can be accurately programmed by an infrared laser.

Supplementary Table 1: 18 XRD peak positions of VO_2 NM with different ψ.

ψ (°)	VO_2 (011)	VO_2 (200)	VO_2 (210)	VO_2 (220)
0	28.40096	37.50716	42.63066	57.5265
18	28.05109	36.6826	41.77429	56.74794
32	27.92002	36.6826	41.77429	56.77655
45	28.02924	36.65977	41.77429	56.71929

Supplementary Table 2: Contribution of different orientations to the stress of VO_2.

Spacings	K (GPa)	M	σ_φ (GPa)	$P(k\ l\ m)$
(0 1 1)	-1.7388	-0.42247	0.73459084	0.3164
(2 0 0)	-1.2248	-0.88122	1.07931826	0.2083
(2 1 0)	-1.0174	-0.8503	0.86509522	0.1721
(2 2 0)	-0.6364	-0.8113	0.51631132	0.3032

Supplementary Table 3: ΔT_{sol} and T_{lum} of different samples with various K and L/L^*.

SW sample K(10^4 m^{-1})-L/L^*	$T_{lum,\ low\ \tau}$ (%)	$T_{lum,\ high\ \tau}$ (%)	ΔT_{sol} (%)
1.45-0.67	59.022	46.539	20.753
1.26-0.67	47.626	26.566	30.684
0.85-0.67	41.824	29.991	22.927
1.26-0.80	53.729	28.602	35.465
1.26-0.87	61.009	29.303	42.142
0-0 (film)	23.901	20.167	12.553

Supplementary Table 4: Comparison of this work with the best-reported experiment works regarding the τ_c, ΔT_{lum}, T_{lum} and ΔT_{sol}.

	τ_c (°C)	ΔT_{lum} (%)	T_{lum} (%)	ΔT_{sol} (%)
This work	48	31.71	61.01	42.14
VO_2 film	68	3.74	23.90	12.55
Doping Mg[22]	61.05	2.7	82.1	4.8
Doping Sr[23]	68	5.1	54.3	5
Doping Sr[23]	68	4.8	50.3	6.5
Doping W[24]	28	1.3	45.1	6.9

Doping F[25]	34.9	2.8	48.7	10.7
Doping Zr[26]	64.3	5.1	60.4	14.1
Doping Ti[27]	68	6.9	53	17.2
Doping Tb[28]	62.5	1	65.9	4.6
Doping Mg+W[22]	35	3.5	81.3	4.3
PU[29]	91.7	5.6	45.6	22.3
Core-shell VO_2-@-SiO_2[30,31]	68	2.9	27.8	13.6
Core-shell $V_xW_{1-x}O_2$-@-SiO_2[32]	25.2	3.1	50.6	14.7
VO_2+TiO_2[33]	68	1.5	61.2	14.6
PNIPAm[13,17]	68	29.9	62.6	34.7
HPC[34]	50	47	56	36
IL-Ni-Cl[5]	68	22.51	55.2	26.5
CLETS[7]	68	6.9	59.2	20.8
NLETS[6]	42	4.65	71	18.2
PVP[35]	68	2.4	43.3	14.1
Freeze-drying[36]	68	1.2	50	14.7
Electrodeposition[37]	68	4.9	38.4	13.9
Mesh printing[38]	68	2.2	43.3	14.9
Nanosphere Lithography[39]	68	0.3	46	13.2
CeO_2/VO_2[40]	68	7	67.5	5.4
TEOS/VO_2[41]	68	4	52.7	16.4
TiO_2/VO_2/SiO_2[42]	58.5	2.9	61.5	6.9
LSAR kirigami[43]	70	30	35.2	37.7

Supplementary Table 5 Building parameters used in the EnergyPlus simulation.

Building type	Small office building
Number of Floors	1
Toral Floor Area	20m×10m
Average Window-to-Wall Ratio	30%
Temperature setting point for HVAC control	Below 21°C for heating/Above 24°C for cooling
Setpoint for lighting on	1000 lux

Supplementary Table 6 Optical information of the window used in the simulation.

	Normal window	VO_2 NM		Rolled-up SW	
States (τ_c=24°C)	-	Cold state	Hot state	Cold state	Hot state
Solar	0.775	0.3527	0.2347	0.724	0.2347

transmittance					
Front side solar reflectance	0.071	0.3112	0.2691	0.219	0.2691
Back side solar reflectance	0.071	0.2357	0.1742	0.239	0.1742
Visible transmittance	0.881	0.3247	0.3038	0.722	0.3038
Front side visible reflectance	0.08	0.194	0.1735	0.181	0.1735
Backside visible reflectance	0.08	0.0829	0.0893	0.16	0.0893
Infrared transmittance	0	0	0	0	0
Front side infrared emissivity	0.84	0.823	0.706	0.792	0.706
Back side infrared emissivity	0.84	0.84	0.84	0.84	0.84

Supplementary Table 7 The annual energy saving of different thermochromic SW in Hong Kong, Shanghai and Albuquerque, respectively.

Thermochromic SW (window-to-wall ratio)		Hong Kong		Shanghai		Albuquerque	
		E_c (MJ/m^2)	E_{saving} (%)	E_c (MJ/m^2)	E_{saving} (%)	E_c (MJ/m^2)	E_{saving} (%)
Rolled-up SW (0.3)		48.05	10.56	26.10	5.69	71.05	13.08
Perovskite/Low E glass[44] (0.375)		27.65	9.1				
Perovskite/Hydrogel[45] (0.385)		25.65	9.2				
Phase change polymer[21] (0.5)		34.09	13.8				
Hydrogel[46] (0.5)		150					
Liquid hydrogel[47] (0.5)	1 mm			14.73	10.7		
	1 cm			25.68	19.2		
VO_2 radiation cooling[48] (0.33)							6.5

Supplementary Note 1: **Fresnel equations for sun incident angle study.**

The Fresnel equations describe the reflection and transmission of light (or electromagnetic radiation in general) when incident on an interface between different optical media:

$$T_s = \frac{n_2}{n_1} t_s^2, \tag{1}$$

$$T_p = \frac{n_2}{n_1} t_p^2, \tag{2}$$

$$t_s = \frac{2cos\theta_1 sin\theta_2}{\sin(\theta_1+\theta_2)}, \tag{3}$$

$$t_p = \frac{2cos\theta_1 sin\theta_2}{\sin(\theta_1+\theta_2)\cos(\theta_1-\theta_2)}, \tag{4}$$

$$n_1 sin\theta_1 = n_2 sin\theta_2. \tag{5}$$

In the equations, T_s is the amplitude transmission for s polarization and T_p is for p polarization. The transmission coefficient t is the ratio of the transmitted wave's complex electric field amplitude to that of the incident wave, for either polarization. The coefficients t is generally different between the s and p polarizations, and even at normal incidence. The θ_1 is incidence angle and θ_2 is angle of refraction.

Supplementary Note 2: **Calculation of initial strain and phase transition strain of VO_2 NMs.**

Supplementary Fig. 5 shows the XRD patterns of VO_2 NMs that are measured by small-angle synchrotron radiation XRD with different incidence angles (ψ), and the specific data are recorded in Supplementary Table 1. It presents that the peak position of VO_2 shifts to a smaller angle with increasing ψ. So, the stress state of NM can be accurately calculated by a series of formula[49] The residual stress σ_φ in the NM is

$$\sigma_\varphi = -\frac{E}{2(1+\nu)} cot\theta_0 \frac{\pi}{180} \frac{\partial(2\theta)}{\partial(sin^2\psi)}, \tag{6}$$

where σ_φ is the stress of a certain crystal orientation (φ) at different ψ, v is the Poisson ratio, E is the Young's modulus of the VO_2 (~140 GPa) and θ is the Bragg angle.

For the sake of clarity, here we define

$$K = -\frac{E}{2(1+\nu)} cot\theta_0, \tag{7}$$

and

$$M = \frac{\partial(2\theta)}{\partial(sin^2\psi)}. \tag{8}$$

Thus, we obtain

$$\sigma_\varphi = K \times M. \tag{9}$$

The XRD scans of polycrystalline VO_2 NMs show the contribution of several orientations, namely (011), (200), (210) and (220). The relative domain population

corresponding to the $(k\ l\ m)$-oriented area, which represents the concentration of different orientation, can be represented as follows[50]:

$$P_{(k\,l\,m)} = \frac{I(k\,l\,m)}{(I(0\ 1\ 1)+I(2\ 0\ 0)+I(2\ 1\ 0)+I(2\ 2\ 0))}, \tag{10}$$

where $I\ (k\ l\ m)$ represents the XRD intensity of the $(k\ l\ m)$ reflection. The domain population $P_{(k\ l\ m)}$ corresponding to (011), (200), (210) and (220) oriented area can therefore be determined by XRD diagrams.

Average stress σ in the NM can thus be calculated by

$$\sigma = \sum_{i=1}^{4} P_{(k\,l\,m)} \sigma_{\varphi}^{(k\,l\,m)}(x) \tag{11}$$

Thus, we propose an approach to determine the residual stress in VO_2 NMs with the help of XRD characterization, and the influence from the substrate temperature can be investigated. Based on the XRD results shown in Supplementary Fig. 5, the calculated stress statuses are given in Supplementary Table 2. The average internal stresses σ of VO_2 NMs are calculated to be 0.7627 GPa deposited at 500 °C. And $\Delta\varepsilon = \Delta\sigma/E =$ 0.7627 GPa/E = 0.5448%.

An account of the relationship between composition, lattice spacing (d), and strain has been given in the literature[51]. The in-plane strain leads to an expansion of lattice planes along the growth direction. The lattice spacing along that is according to the substrate, $d^{\perp}(y)$, is related to the bulk lattice spacing $d(y)$ of the rutile VO_2. Once the unstrained lattice parameter a of the film is known, the strain in the film parallel and perpendicular to the growth direction can be calculated using

$$\varepsilon^{\perp} = \frac{d^{\perp}(y) - d(y)}{d(y)} \tag{12}$$

$$\varepsilon^{/\!/} = \frac{d^{/\!/}(x) - d(x)}{d(x)} \tag{13}$$

Here, $d^{\perp}(y)$ represents the lattice spacing of Y and $d^{/\!/}(x)$ represents the lattice spacing of Y, that is shown in Supplementary Fig. 7. Since the strain producing deformation mainly originates from the direction parallel to the growth plane (X direction), the phase transition strain ($\Delta\varepsilon$) can be considered as

$$\Delta\varepsilon = \varepsilon^{/\!/}{}_{\text{high}\,\tau} - \varepsilon^{/\!/}{}_{\text{low}\,\tau} = \frac{d^{/\!/}(x)_{\text{high}\,\tau} - d^{/\!/}(x)_{\text{low}\,\tau}}{d(x)} \tag{14}$$

Supplementary Note 3: **Calculation of strain with different rolled-up SW sample**

The relation between the Raman shifts and the initial strain gradient ($\Delta\varepsilon$) generated in rolled-up SW can be expressed as:

$$\Delta\omega = -b_{uni} \times \Delta\varepsilon \tag{15}$$

where $\Delta\omega$ represents the Raman peak positions shifts of ω_1 and ω_2 mode according to the Supplementary Fig. 21, and b_{uni} = 791 is the strain-shift coefficient under purely uniaxial stress situations[1,52].

To quantitatively describe the radius variation as a function of $\Delta\varepsilon$, we refer to the linear strain theory model used for bilayer systems. According to this theory model, we

divide the NM into two regions. The lower VO_2 is a relaxed layer that is close to the interface of the deposited layer, as well as the upper VO_2 region is a strained layer. We assume that the elastic coefficients are equal in the two regions. The K of rolled-up microstructure can be calculated by[53]

$$K = \frac{6\varepsilon(1+v)d_{strain}d_{relax}}{(d_{strain}+d_{relax})^3} \quad (16)$$

where ε is the strain in the VO_2 NM and the v is the Poisson ratio. The d_{strain} is always equal to 3 nm and the d_{relax} values are 57 nm, 97 nm and 137 nm in different samples.

Supplementary Note 4: **Calculation methods of ΔT_{sol} and T_{lum}.**

Integral visible transmittance ($\bar{T}_{vis}$) (380-780 nm), near infrared transmittance ($\bar{T}_{NIR}$) and solar transmittance ($\bar{T}_{sol}$) (350-2600 nm) are calculated by equations:

$$\bar{T}_{vis} = \int \Phi_{lum}(\lambda)T(\lambda)d(\lambda) / \int \Phi_{lum}(\lambda)\, d(\lambda) \quad (17)$$

$$\bar{T}_{sol} = \int \Phi_{sol}(\lambda)T(\lambda)d(\lambda) / \int \Phi_{sol}(\lambda)\, d(\lambda) \quad (18)$$

$$\bar{T}_{NIR} = \bar{T}_{sol} - \bar{T}_{vis} \quad (19)$$

where $\Phi_{lum}(\lambda)$ is standard efficiency function for photopic vision, indicating the sensitivity of the human eye to different wavelengths in the visible light region. $\Phi_{sol}(\lambda)$ is the solar irradiance spectrum for an air mass of 1.5, which corresponds to the sun sanding 37° above the horizon. *T(λ)* represents the transmittance at wavelength λ. Solar modulation (ΔT_{sol}) is an important index to evaluate the thermochromic property of VO_2, which is calculated by:

$$\Delta T_{sol} = \bar{T}_{sol,low\,\tau} - \bar{T}_{sol,high\,\tau} \quad (20)$$

where low τ and high τ represent 20 °C and 80 °C, respectively.

Supplementary Note 5: **Simulation ΔT_{sol} as a function of K and *L/L**.**

The transmittance simulation was performed using commercial software Essential Macleod (Thin Film Center Inc, USA) based on the multilayer optical theory with a transfer-matrix method. The optical constants (*n*, *k*) of VO_2 nanomembrane in low and high τ were taken from literature in the range of 350-2500 nm[54]. The refractive index of the quartz substrate was 1.5. The thickness of VO_2 NM was fixed at 100 nm when simulating the spectra transmittance *T(λ)* at low and high τ.

At high τ, the rolled-up SW maintained flat on the quartz substrate, and its transmittance $\bar{T}_{sol,high\,\tau,rolled-up}$ is approximately equal to the transmittance of the VO_2 NM $\bar{T}_{sol,high\,\tau,NM}$:

$$\bar{T}_{sol,high\,\tau,rolled-up} = \bar{T}_{sol,high\,\tau,NM} \quad (21)$$

At low τ, the rolled-up SW shifts to rolled state, and the relationship between the transmittances and the geometric parameters can be approximately described as:

$$\bar{T}_{sol,low\,\tau,rolled-up} = \bar{T}_{sol,low\,\tau,NM} \cdot \left[1 - L \cdot (L - 1/k)/L^{*2}\right] + L \cdot (L - 1/k)/L^{*2} \quad (22)$$

As mentioned in equation 20, solar modulation ability of rolled-up SW can be calculated:

$$\Delta\bar{T}_{sol,rolled-up} = \bar{T}_{sol,low\,\tau,rolled-up} - \bar{T}_{sol,high\,\tau,rolled-up} \quad (23)$$

Supplemental references

[1] Atkin, J.M. et al. Strain and temperature dependence of the insulating phases of VO_2 near the metal-insulator transition. *Phys. Rev. B* **85**, 020101 (2012).

[2] Chen, Y. et al. VO_2/Nickel-bromine-ionic liquid composite film for thermochromic application. *Sol. Energy Mater. Sol. Cells* **196**, 124-130 (2019).

[3] Kakiuchid, H., Tazawa, M., Yoshimura, A. & Ogiwara, A. Thermal control of transmittance/diffraction states of holographic structures composed of polymer and liquid crystal phases. *Sol. Energy Mater. Sol. Cells* **94**, 1747-1752 (2010).

[4] Liang, X. et al. Dual-band modulation of visible and near-infrared light transmittance in an all-solution-processed hybrid micro-nano composite film. *ACS Appl. Mater. Interfaces* **9**, 40810-40819 (2017).

[5] Zhu, J. et al. Composite film of vanadium dioxide nanoparticles and ionic liquid-nickel-chlorine complexes with excellent visible thermochromic performance. *ACS Appl. Mater. Interfaces* **8**, 29742-29748 (2016).

[6] Zhu, J. et al. Hybrid films of VO_2 nanoparticles and a nickel (II)-based ligand exchange thermochromic system: Excellent optical performance with a temperature responsive color change. *New J. Chem.* **41**, 830-835 (2017).

[7] Zhu, J. et al. Solar-thermochromism of a hybrid film of VO_2 nanoparticles and CoII-Br-TMP complexes. *RSC Adv.* **6**, 67396-67399 (2016).

[8] Liu, S. et al. Near-Infrared-Activated Thermochromic perovskite smart windows. *Adv. Sci.* **9**, 2106090 (2022).

[9] Lee, H Y. et al. Thermochromic ionogel: a new class of stimuli responsive materials with super cyclic stability for solar modulation. *Chem. Mater.* **29**, 6947-6955 (2017).

[10] Halder, A. et al. Exploring thermochromic behavior of hydrated hybrid perovskites in solar cells. *J Phys. Chem. Lett.* **6**, 3180-3184 (2015).

[11] Zhang, Y. et al. Perovskite thermochromic smart window: Advanced optical properties and low transition temperature. *Appl. Energy* **254**, 113690 (2019).

[12] Liu, S. et al. Organic hybrid perovskite ($MAPbI_{3-x}Cl_x$) for thermochromic smart window with strong optical regulation ability, low transition temperature, and narrow hysteresis width. *Adv. Funct. Mater.* **31**, 2010426 (2021).

[13] Zhou, Y., Cai, Y., Hu, X. & Long, Y. Temperature-responsive hydrogel with ultra-large solar modulation and high luminous transmission for “smart window” applications. *J. Mater. Chem. A* **2**, 13550-13555 (2014).

[14] Li, X., Liu, C., Feng, S. & Fang, N.X. Broadband light management with thermochromic hydrogel microparticles for smart windows. *Joule* **3**, 290-302 (2019).

[15] Yang, Y., Zhou, Y., Chiang, F.B.Y. & Long, Y. Temperature-responsive hydroxypropylcellulose based thermochromic material and its smart window application. *RCS Adv.* **6**, 61449-61453 (2016).

[16] Zhou, Y. et al. Fully printed flexible smart hybrid hydrogels. *Adv. Funct. Mater.* **28**, 1705365 (2018).

[17] Zhou, Y., Cai, Y., Hu, X. & Long, Y. VO_2/hydrogel hybrid nanothermochromic material with ultra-high solar modulation and luminous transmission. *J. Mater.*

Chem. A **3**, 1121-1126 (2015).
[18] Xu, Z. et al. Sunlight-induced photo-thermochromic supramolecular nanocomposite hydrogel film for energy-saving smart window. *Sol. RRL* **2**, 1800204 (2018).
[19] Wu, M., Shi, Y., Li, R. & Wang, P. Spectrally selective smart window with high near-infrared light shielding and controllable visible light transmittance. *ACS Appl. Mater. Interfaces* **10**, 39819-39827 (2018).
[20] Xie, Y. et al. A phase-changing polymer film for broadband smart window applications. *Macromol. Rapid Commun.* **41**, 2000290 (2020).
[21] Li, D. et al. Deformable thermo-responsive smart windows based on a shape memory polymer for adaptive solar modulations. *ACS Appl. Mater. Interfaces* **13**, 61196-61204 (2021).
[22] Wang, N., Liu, S., Zeng, X.T., Magdassi, S. & Long, Y. Mg/W-codoped vanadium dioxide thin films with enhanced visible transmittance and low phase transition temperature. *J. Mater. Chem. C* **3**, 6771-6777 (2015).
[23] Dietrich, M.K. et al. Influence of doping with alkaline earth metals on the optical properties of thermochromic VO_2. *J. Appl. Phys.* **117**, 185301 (2015).
[24] Hu, L., et al. Porous W-doped VO_2 films with simultaneously enhanced visible transparency and thermochromic properties. *J. Solgel Sci. Technol* **77**, 85-93 (2016).
[25] Dai, L. et al. F-doped VO_2 nanoparticles for thermochromic energy-saving foils with modified color and enhanced solar-heat shielding ability. *Phys. Chem. Chem. Phys.* **15**, 11723-11729 (2013).
[26] Shen, N. et al. The synthesis and performance of Zr-doped and W-Zr-codoped VO_2 nanoparticles and derived flexible foils. *J. Mater. Chem. A* **2**, 15087-15093 (2014).
[27] Chen, S. et al. The visible transmittance and solar modulation ability of VO2 flexible foils simultaneously improved by Ti doping: An optimization and first principle study. Phys. Chem. Chem. Phys. 15, 17537-17543 (2013).
[28] Wang, N. et al. Terbium-doped VO_2 thin films: Reduced phase transition temperature and largely enhanced luminous transmittance. *Langmuir* **32**, 759-764 (2016).
[29] Chen, Z. et al. Fine crystalline VO_2 nanoparticles: Synthesis, abnormal phase transition temperatures and excellent optical properties of a derived VO_2 nanocomposite foil. *J. Mater. Chem. A* **2**, 2718-2727 (2014).
[30] Gao, Y. et al. Enhanced chemical stability of VO_2 nanoparticles by the formation of SiO_2/VO_2 core/shell structures and the application to transparent and flexible VO_2-based composite foils with excellent thermochromic properties for solar heat control. *Energy Environ. Sci.* **5**, 6104-6110 (2012).
[31] Zhou, Y. et al. Surface plasmon resonance induced excellent solar control for $VO_2@SiO_2$ nanorods-based thermochromic foils. *Nanoscale* **5**, 9208-9213 (2013).
[32] Zhu, J. et al. Vanadium dioxide nanoparticle-based thermochromic smart coating: High luminous transmittance, excellent solar regulation efficiency, and near room temperature phase transition. *ACS Appl. Mater. Interfaces* **7**, 27796-27803 (2015).
[33] Chen, Z., Cao, C., Chen, S., Luo, H. & Gao, Y. Crystallised mesoporous TiO_2(A)-VO_2(M/R) nanocomposite films with self-cleaning and excellent thermochromic

properties. *J. Mater. Chem.* A **2**, 11874-11884 (2014).
[34] Yang, Y.S., Zhou, Y., Chiang, F. & Long, Y. Tungsten doped VO_2/microgels hybrid thermochromic material and its smart window application. *RSC Adv.* **7**, 7758-7762 (2017).
[35] Kang, L. et al. Nanoporous thermochromic VO_2 films with low optical constants, enhanced luminous transmittance and thermochromic properties. *ACS Appl. Mater. Interfaces* **3**, 135-138 (2011).
[36] Cao, X. et al. Nanoporous thermochromic VO_2(M) thin films: Controlled porosity, largely enhanced luminous transmittance and solar modulating ability. *Langmuir* **30**, 1710-1715 (2014).
[37] Liu, C., Long, Y., Magdassi, S. & Mandler, D. Ionic strength induced electrodeposition: A universal approach for nanomaterial deposition at selective areas. *Nanoscale* **9**, 485-490 (2017).
[38] Lu, Q. et al. Periodic micro-patterned VO_2 thermochromic films by mesh printing. *J. Mater. Chem. C* **4**, 8385-8391 (2016).
[39] Ke, Y. et al. Controllable fabrication of two-dimensional patterned VO_2 nanoparticle, nanodome, and nanonet arrays with tunable temperature-dependent localized surface plasmon resonance. *ACS Nano* **11**, 7542-7551 (2017).
[40] Koo, H. et al. The effect of CeO_2 antireflection layer on the optical properties of thermochromic VO_2 film for smart window system. *J. Mater. Eng. Perform* **23**, 402-407 (2014).
[41] Liu, C. et al. Index-tunable anti-reflection coatings: Maximizing solar modulation ability for vanadium dioxide-based smart thermochromic glazing. *J. Alloys Compd.* **731**, 1197-1207 (2018).
[42] Chen, Z. et al. VO_2- based double-layered films for smart windows: Optical design, all-solution preparation and improved properties. *Sol. Energy Mater. Sol. Cells* **95**, 2677-2684 (2011).
[43] Ke, Y. et al. Adaptive thermochromic windows from active plasmonic elastomers. *Joule* **3**, 858-871 (2019).
[44] Liu, S. et al. Near-infrared-activated thermochromic perovskite smart windows. *Adv. Sci.* **9**, 2106090 (2022).
[45] Meng, Y. et al Building-integrated photovoltaic smart window with energy generation and conservation. *Appl. Energy* **324**, 119676 (2022).
[46] Lin, C. et al. All-weather thermochromic windows for synchronous solar and thermal radiation regulation. *Sci. Adv.* **8**, eabn7359 (2022).
[47] Zhou, Y. et al. Liquid thermo-responsive smart window derived from hydrogel. *Joule* **4**, 2458-2474 (2020).
[48] Ke, Y. et al. On-demand solar and thermal radiation management based on switchable interwoven surfaces. *ACS Energy Lett.* **7**, 1758-1763 (2022).
[49] Zheng, X., Li, J. & Zhou, Y. X-ray diffraction measurement of residual stress in PZT thin films prepared by pulsed laser deposition. *Acta Materialia* **52**, 3313-3322 (2004).
[50] Sakashita, Y., Ono, T., Segawa, H., Tominaga, K. & Okada, M. Preparation and electrical properties of MOCVD-deposited PZT thin films. *J. Appl. Phys.* **69**, 8352

(1991).
[51] Rosenauer, A. Transmission Electron Microscopy of Semiconductor Nanostructures (Springer Berlin, Heidelberg, 2003).
[52] Süess, M.J. et al. Analysis of enhanced light emission from highly strained germanium microbridges. *Nat. Photonics* **7**, 466-472 (2013).
[53] Songmuang, R., Deneke, C. & Schmidt, O.G. Rolled-up micro- and nanotubes from single-material thin films. *Appl. Phys. Lett.* **89**, 223109 (2006).
[54] Long, S. et al. Self-template synthesis of nanoporous VO_2-based films: localized surface plasmon resonance and enhanced optical performance for solar glazing application. *Appl. Mater. Interfaces* **11**, 22692-22702 (2019).
