# Supplementary material for: Self-rolling of vanadium dioxide nanomembranes for enhanced multi-level solar modulation
Source: Nat Commun. 2022 Dec 19;13:7819. doi: 10.1038/s41467-022-35513-w (PMC9763237; doi:10.1038/s41467-022-35513-w)
Supplement: Supplementary file 3 — Description of Additional Supplementary Files [file 41467_2022_35513_MOESM3_ESM.pdf]

Description of Additional Supplementary Files

File Name: Supplementary Movie 1.mp4
Description: Deformation of rolled-up SW during heating.

File Name: Supplementary Movie 2.mp4
Description: Deformation of rolled-up SW during heating recorded by optical microscope.

File Name: Supplementary Movie 3.mp4
Description: Rolled-up SW as lens protective cover during heating (visible light range).

File Name: Supplementary Movie 4.mp4
Description: Rolled-up SW as lens protective cover during heating (NIR range).
